# A study of organic acid production in contrasts between two phosphate solubilizing fungi: *Penicillium oxalicum* and *Aspergillus niger*

**DOI:** 10.1038/srep25313

**Published:** 2016-04-29

**Authors:** Zhen Li, Tongshuo Bai, Letian Dai, Fuwei Wang, Jinjin Tao, Shiting Meng, Yunxiao Hu, Shimei Wang, Shuijin Hu

**Affiliations:** 1 College of Resources and Environmental Sciences, Nanjing Agricultural University, Nanjing, Jiangsu 210095, China; 2 Department of Plant Pathology, North Carolina State University, Raleigh, NC 27695, USA

## Abstract

Phosphate solubilizing fungi (PSF) have huge potentials in enhancing release of phosphorus from fertilizer. Two PSF (NJDL-03 and NJDL-12) were isolated and identified as *Penicillium oxalicum* and *Aspergillus niger* respectively in this study. The quantification and identification of organic acids were performed by HPLC. Total concentrations of organic acids secreted by NJDL-03 and NJDL-12 are ~4000 and ~10,000 mg/L with pH values of 3.6 and 2.4 respectively after five-days culture. Oxalic acid dominates acidity in the medium due to its high concentration and high acidity constant. The two fungi were also cultured for five days with the initial pH values of the medium varied from 6.5 to 1.5. The biomass reached the maximum when the initial pH values are 4.5 for NJDL-03 and 2.5 for NJDL-12. The organic acids for NJDL-12 reach the maximum at the initial pH = 5.5. However, the acids by NJDL-03 continue to decrease and proliferation of the fungus terminates at pH = 2.5. The citric acid production increases significantly for NJDL-12 at acidic environment, whereas formic and oxalic acids decrease sharply for both two fungi. This study shows that NJDL-12 has higher ability in acid production and has stronger adaptability to acidic environment than NJDL-03.

Low mobility of phosphorus is one of the primary limiting factors for crop production in soils, which is attributed to its low solubility, chemical fixation, and complex chelation^1^,^2^. In addition, P is one of the “minor” elements on the Earth (0.1% of total), and current global phosphate rock could be depleted within 50–100 years due to increasing demand and limited P mineral reserves^3^. Improving P release from phosphate minerals is hence a critical issue in agriculture. P mineral, i.e., geological apatite, is the primary source of P fertilizer. Acidic environment can enhance the solubility of P minerals significantly^1^,^4^,^5^,^6^, which is a feasible pathway to improve the P release from phosphate minerals.

Many phosphorous solubilizing microbes (PSM), including species of bacteria, fungi, and actinomyces, have the ability to produce organic acids^7^. They have also been shown to enhance the solubilization of insoluble P compounds^8^. Therefore, they have been widely applied to increase P uptake and crop yield^9^. Some of these microbes promote plant growth by secreting indole acetic acid (IAA) and siderophore, in addition to the organic acids^10^. Compared to bacteria, phosphorous solubilizing fungi (PSF) have ten times higher in their ability to secrete organic acids^7^. The pH values in the culture medium of PSF can be decreased to as low as 1 to 2^5^. Therefore, PSF are considered as primary candidates in the pool of PSM^11^.

The ability of organic acids secretion is basically determined by genes, but can also be affected by environmental conditions. For example, carbon and nitrogen could affect the types of organic acids and phosphate solubilizing^12^. A high C/P will increase organic acids production^13^, and various C/N and N/P ratios affect growth of microbes^14^. PSM could solubilize P minerals via various mechanisms. For examples, microbial respiration and NH_4_^+^ ions can also enhance release of mobile inorganic P through releasing protons^6^. Additionally, chelation is usually assumed to be an efficient mechanism of P solubility^14^.

The isolation of PSF from soils had been widely investigated, especially for filamentous fungi of *Penicillium,* and *Aspergillus*^15^,^16^,^17^. The phosphate-solubilizing ability is generally associated to the release of organic acids with the decrease in the pH values^18^. However, that how the acidity in the environment affects PSF’s activity has not been well documented. In addition, the detailed comparison between typical PSF, e.g., *P. oxalicum* and *A. niger*, has not been deeply investigated. For example, the comparison in organic acids secretion (quantification and identification) among PSF under various acidic environments is still unclear.

The aim of this study is to investigate the acid secretion of *P. oxalicum* (CGMCC No.11061, NJDL-03) and *A. niger* (CGMCC No.11544, NJDL-12), which were isolated from soils in our lab. The two fungi have the highest ability in secretion of organic acids in the pool (screened in our lab) of *Penicillium* and *Aspergillus* respectively.

## Results

### Identification of NJDL-03 and NJDL-12

**Figure 1:**
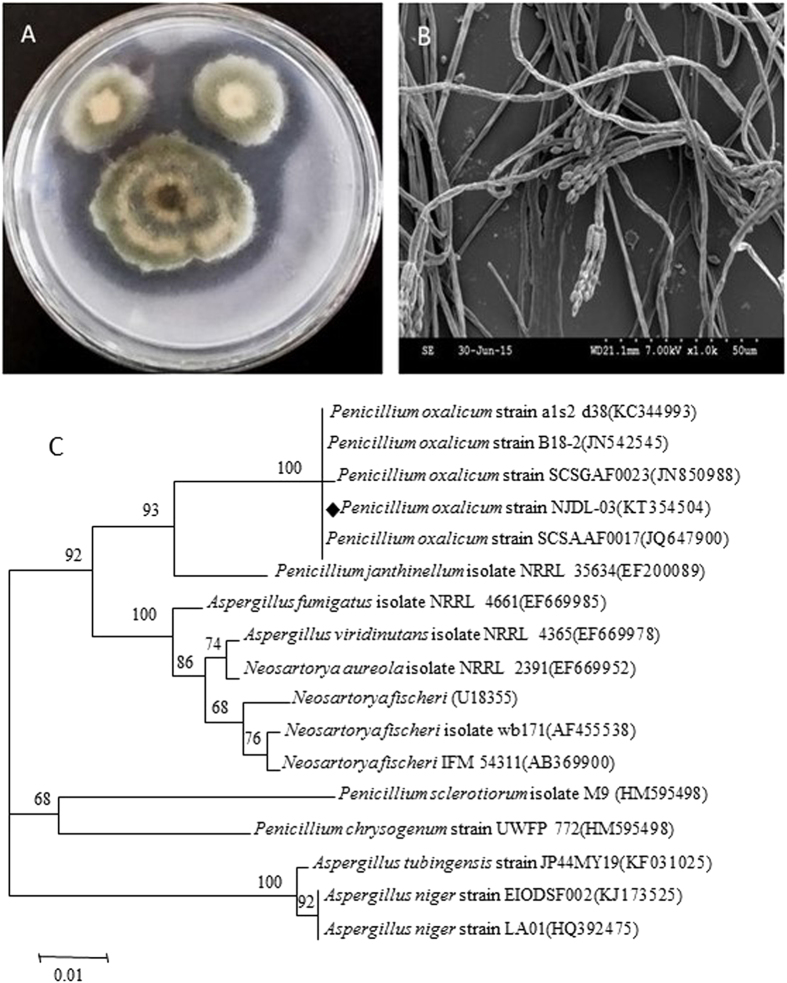
Identification of NJDL-03. (**A**) image of the colony; (**B**) broom-shape conidiophores of NJDL-03; (**C**) phylogenetic tree of the ITS sequences of NJDL-03. It is identified as *P. oxalicum* (labeled as diamond).

**Figure 2:**
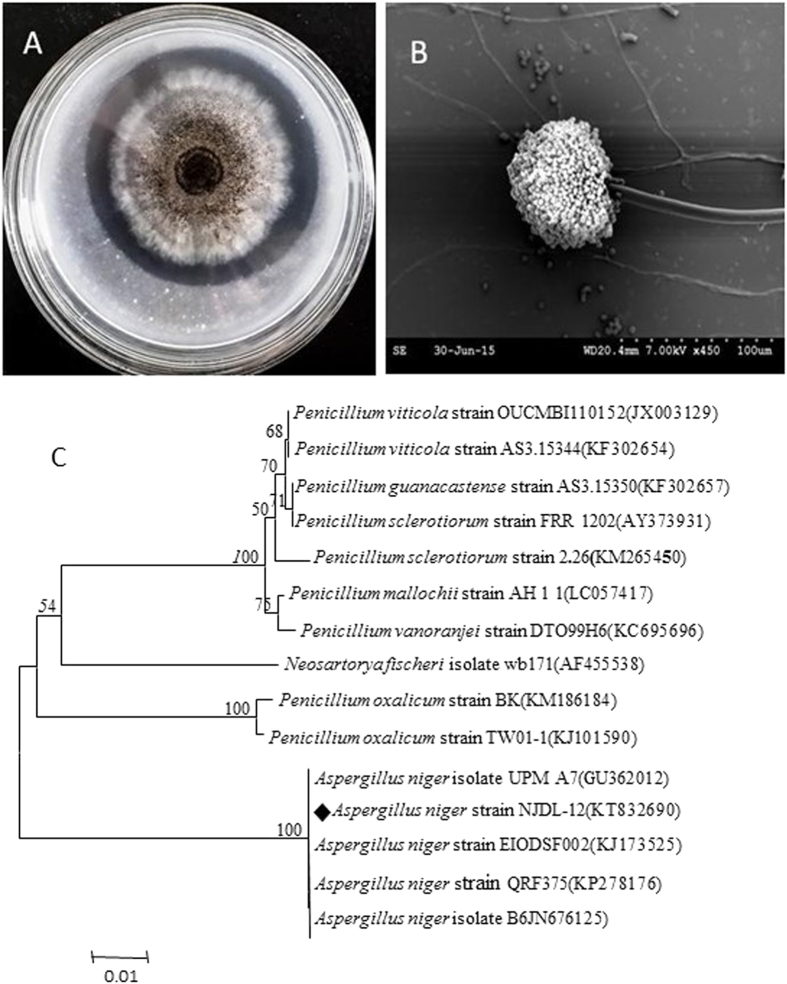
Identification of NJDL-12. (**A**) image of the colony; (**B**) head-shape sporangium of NJDL-12; (**C**) phylogenetic tree of the ITS sequences of NJDL-12. It is identified as *A. niger* (labeled as diamond).

A clear zone of dissolved phosphate and colony morphology of strains in Pikovskaya’s agar plate show the high phosphate-solubilizing ability of NJDL-03 and NJDL-12 (Figs 1A and 2A). Characteristic morphology of the hypha, spores, and conidiophores in petri dishes were shown in Figs 1 and 2. In Fig. 1A,B, NJDL-03 has typical broom shape-conidiophores with green conidia. ITS rRNA gene sequence was applied to identify the strain NJDL-03, and it was identified as *P. oxalicum* (Fig. 1C).

Strain NJDL-12 has typical “ball-shape” sporangium, and the black conidia in conidiophores can be identified (Fig. 2B). ITS rRNA gene sequence of the strain NJDL-12 confirms it as *A. niger* (Fig. 2C).

### Mycelial acidity and biomass of NJDL-03 and NJDL-12

Table 1 shows that the pH values of the culture medium (initial pH = 6.5) were significantly decreased in the culture medium. After five days, the pH value of NJDL-12 decreased to 2.4 while the NJDL-03 decreased to 3.6, indicating that NJDL-12 has higher ability in enhancing acidity than NJDL-03 (Table 1).

**Figure 3:**
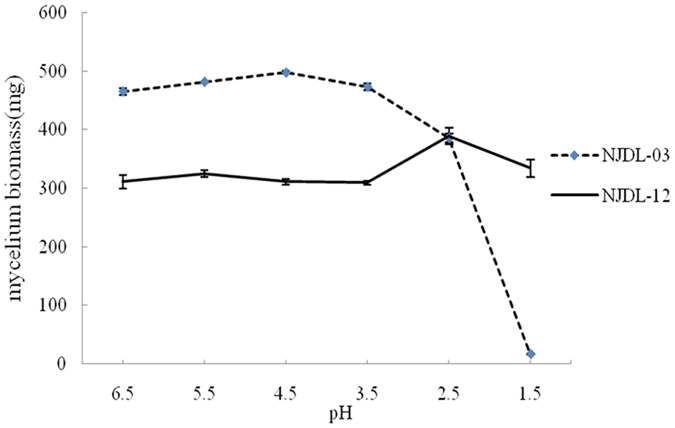
Biomass after five-days culture in the medium with various initial pH values.

Strain NJDL-03 and NJDL-12 were then cultured in medium with initial pH values of the medium ranged from 6.5 to 1.5. Mycelial biomass is a significant parameter to directly evaluate the growth of fungi. The two fungi show distinct biomass changes under various acidic environments (Table 1). NJDL-12 shows better resistance to the acidic environment, whereas the proliferation of NJDL-03 terminates at pH = 2.5 (no mycelium pellet and few dispersive mycelia can be identified under microscope). The maximum biomass of NJDL-12 was 389 mg in medium when initial pH = 2.5, and the growth was constant in acidic environments (Fig. 3). To the contrast, the biomass of NJDL-03 arrived at a peak value of 497 mg when pH = 4.5.

### Organic acid secretion of NJDL-03 and NJDL-12

**Figure 4:**
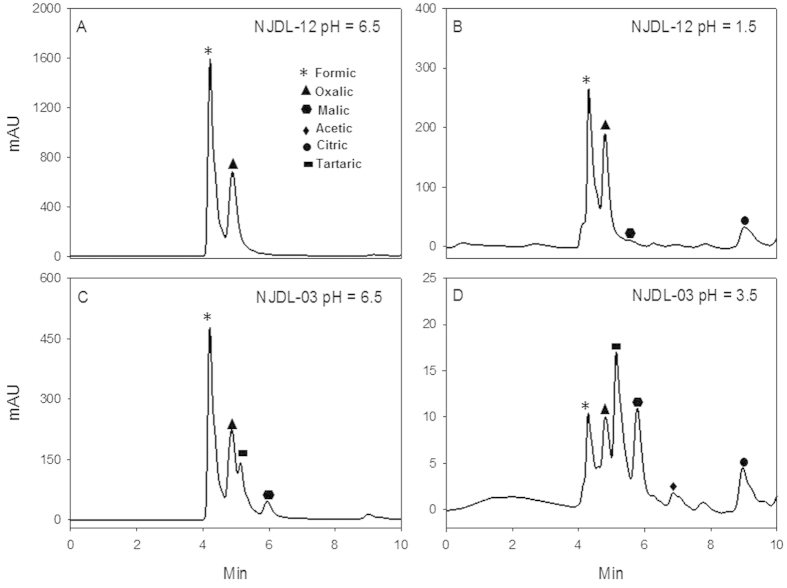
Diagrams of the secreted organic acids analyzed by HPLC. The acids were produced by NJDL-03 and NJDL-12 at initial pH values of 6.5 and 1.5 respectively.

Several organic acids with low molecular weight, e.g., oxalic, formic, tartaric, malic, acetic, and citric acids, were secreted by the two PSF (Table 2). Lactic and indoleacetic acids were not detected in culture medium. The major secreted organic acids of NJDL-12 were oxalic and formic acids, whereas NJDL-03 has oxalic, formic, and tartaric acids. Citric acid was only detected at low pH environment (Fig. 4 and Table 2). Although the dominant acids in NJDL-03 are also formic and oxalic acids at initial pH = 6.5, its quantity of acids for is significantly lower than those from NJDL-12 (Table 2).

With the increase of the original acidity in the culture medium, the concentration of each single organic acid secreted by the NJDL-03 and NJDL-12 varies. NJDL-12 has a descending trend for concentration of the organic acids, i.e., oxalic and formic acid decreased from 2353 to 437 mg/L and from 7656 to 1710 mg/L respectively. NJDL-03 has a similar trend, i.e., from 902 to 0 mg/L and from 1546 to 34 mg/L respectively (Table 2). In addition, tartaric acid secreted by NJDL-03 was also sharply decreased from 1188 to 55 mg/L. Citric acid secretion for NJDL-12 has a transition point at pH = 2.5, i.e., its concentration doubles from pH = 3.5 to 2.5 and continues to increase. Its concentration is ~six times higher at pH = 1.5 than that at pH = 6.5 (Table 2).

Concentration of organic acids secreted by the NJDL-12 reaches the maximum at pH = 5.5. To the contrast, NJDL-03 descends its secretion continuously down to pH = 3.5 (Fig. 4 and Table 2). As the initial pH values of the medium decreasing, the total quantity of the organic acid secreted by NJDL-12 decreases, and it reaches the minimum at pH = 1.5 (Fig. 4). Additionally, malic acid and acetic acid secreted by NJDL-12 are found in acidity environment (Fig. 4).

## Discussion

The five-days culture in this study show that both NJDL-03 and NJDL-12 have impressive potentials in enhancing P release within such a short time. The organic acids secreted by fungi contribute to their solubilizing ability. To investigate the mechanisms, there are three factors that should be addressed, i.e., pH values, types of organic acids, and feedback to environmental acidity. In the present study, *P. oxalicum* NJDL-03 and *A. niger* NJDL-12 were isolated from the soils and were identified as the fungi with prominent solubilizing ability in the pools of *Penicillium* and *Aspergillus* (in our lab) respectively.

The *A. niger* NJDL-12 shows higher solubilizing ability compared to *P. oxalicum* NJDL- 03 as it has higher organic acids secretion (see total quantity in Table 2). Considering the lower biomass of NJDL-12 compared to that of NJDL-03 (Table 1), NJDL-12 shows high efficiency in acid secretion per unit of biomass. The pH reduction can be attributed to diffusion of various organic acids secreted by the PSF^19^. In soils, the advantages of organic acids compared to inorganic acids are: 1) few negative effects to soil quality, e.g., salinization; 2) release of more H^+^ due to their low acidity constants (at the same pH as inorganic acids).

A serial of organic acids have various acidity constants, which determine their ability in changing acidity of the environments. The acidity constants for the major organic acids secreted by the two PSF are: Kα_1_ = 6.5 × 10^−2^ (oxalic acid), Kα = 1.78 × 10^−4^ (formic acid), Kα_1_ = 9.2 × 10^−4^ (tartaric acid), and Kα_1_ = 7.4 × 10^−4^ (citric acid). Therefore, oxalic acid dominates the acidity in the medium as its first degree ionization constant is about ~100 times higher than those of formic, tartaric, and citric acids. Therefore, NJDL-12 has the significantly higher ability in solubilizing P minerals, not only due to the total quantity of organic acids but also for the high oxalic acid secretion (Table 2). Furthermore, oxalic and formic acids are more prominent (in proportion of the total concentration) for NJDL-12 (~90%) than NJDL-03 (50%). Therefore, it is more convenient to evaluate the roles of organic acids for NJDL-12 in the future usage, e.g., biofertilizer based on *A. niger*.

The acidity has significant influence to activities of microorganisms. Two fungal species in this study, *A. niger* (NJDL-12) and *P. oxalicum* (NJDL-03), were all eosinophilic fungi, and their growth was relatively good in acidic environment (pH = 3~4), compared to other fungi or bacteria. However, under the extreme acidic condition (pH < 3), only the biomass of NJDL-12 increases, and its secretion of citric acid starts to dominate the organic acids secretion.

Most of low-molecular weight organic acids are presumably derived from the TCA (Tricarboxylicacidcycle acid) cycle^20^. The prominent oxalate production in fungi occurs by a process called glyoxylate oxidation^20^,^21^,^22^. And oxalic acid is biosynthesized from glucose catalyzed by cytosolic oxaloacetase, with hydrolysis of oxaloacetate to oxalate and acetate^21^. The accumulation of citric acid for *A. niger* at low pH values has also been proposed in previous research^20^,^22^,^23^,^24^. *A. niger* metabolizes glucose and produces citric acid. When the pH value decreases to ~2.0, it causes aconitate hydratase inactive, and TCA cycle is blocked. Then, the massive citric acid accumulation is initiated. At the same time, NAD (Nicotinamide Adenine Dinucleotide) produced by glycolytic pathway was transmitted through the respiratory chain inhibited by SHAM (Salicylhydroxamic acid), to ensure the balance of oxidation reduction potential in *A. Niger*^24^,^25^.

## Conclusions

*A. niger* (NJDL-12) shows higher ability in secreting organic acids and higher adaptability to acidic environments, compared to *P. oxalicum* (NJDL-03). Oxalic acid dominates the acidity (from the fungi) due to its high concentration and high acidity constant. Production of citric acid from A. *niger* is significantly enhanced under acidic environments. To apply PSF in agricultural soils or biofertilizer for enhancing P release, *A. niger* is a better candidate than *P. oxalicum*.

## Materials and Methods

### Isolation and identification of phosphate-solubilizing fungi

Soil samples were collected from the maize rhizosphere (*P. oxalicum*) and soybean rhizosphere (*A. niger*) of Nanjing, China. The maize and soybean roots were shaken carefully inside plastic bags in order to separate the soil from the roots. The rhizosphere soil samples were serially diluted and inoculated on modified Pikovskaya’ s^26^,^27^ agar medium containing 5 g/L insoluble inorganic forms of P (tricalcium phosphate). Petri plates were incubated at 28 °C for 10 days. Filamentous fungi colonies (coded as NJDL-03 from maize rhizosphere and NJDL-12 from soybean rhizosphere) with the larger halos were picked and purified by repeated culturing in the medium of potato dextrose agar (PDA) at 28 °C. The ability of phosphate-solubilizing was evaluated on Pikovskaya’s plate according to the size of the circle of dissolved phosphorus.

The conidiophore shapes of the two strains were observed under scanning electron microscope. Genomic DNA of NJDL-03 and NJDL-12 were extracted respectively after two-days culturing, as described in the previous literature^28^. Its internal transcribed spacer (ITS) region was amplified using a semi-nested PCR protocol^29^, universal primers ITS1 (5′- TCCGTAGGTGAACCTGCGG-3′). ITS4 (5′-TCCTCCGCTTATTGATATGC-3′) was applied for amplification round. The ITS rRNA gene was sequenced and searched in GenBank. Then, the closely related sequences were download, and all the selected sequences were aligned using the ClustalX 1.83^30^. Finally, a neighbor joining phylogenetic tree was constructed by the MEGA 5.0 (Center of Evolutionary Functional Genomics Biodesign Institute, Arizona State University).

### Biomass of fungi under different pH conditions

Strain NJDL-12 and NJDL-03 were prepared by growing plate cultures on PDA at 28 °C for 5 d to form sporulation respectively. Plates were drenched with sterile distilled water, and spores were carefully freed from the culture surface with a fine artist’s brush. The suspension was then filtered through three layers of sterile cheesecloth to eliminate mycelial fragments. The conidia concentration was determined by haemacytometer, and adjusted to 10^7^ cfu ml^−1^ by dilution with 0.85% sterile saline. The potato dextrose liquid medium was precisely regulated to pH 1.5, 2.5, 3.5, 4.5, 5.5 and 6.5 (CK) with hydrochloric acid, and sterilized at 121 °C for 20 min. The 1 ml spore suspension of NJDL-12 and NJDL-03 were inoculated in 100 ml triangular flasks with various acidity. These flasks were incubated at 28 °C for five days under shaking. Then the culture medium was filtered through 0.22 μm membrane, and the mycelium was weighed after drying 10 h at 80 °C. The weight of mycelium was dried at 80 °C for 24 h and measured. All experiments were repeated three times.

### Secretion of organic acids under different pH conditions

After five-days incubation, the pH values of the filtered medium were measured. Then, the contents of the organic acids were analyzed by high performance liquid chromatography (HPLC). Eight organic acids, oxalic, tartaric, formic, acetic, lactic, malic, citric, and indoleacetic acids with 1000 mg/L (oxalic acid 300 mg/L) were prepared for acid identification by HPLC. The standard solution of each organic acid was diluted into 1000, 500, 250, 125, 62.5, 31.25, and 0 mg/L, respectively.

All experiments were repeated three times. All samples were obtained and experiments were performed in accordance with relevant guidelines and regulations.

### Instrumentation

#### The PCR amplification

The PCR amplification was performed by using the thermal cycler (Bio-Rad S1000, USA) with the following cycling parameters: initial denaturation step at 94 °C for 5 mins, 30 cycles (denaturation at 94 °C for 1 min, annealing at 52 °C for 30 s, extension at 72 °C for 1.5 mins), and a final extension at 72 °C for 10 mins.

#### Field-emission scanning electron microscopy (FE-SEM)

FE-SEM was applied using a Hitachi S-3000N system. Fungal materials were fixed with 2.5% glutaraldehyde, and were washed with phosphate buffer for 3 times (10 minutes each time). Then, they were dehydrated in a series of alcohol (50%, 70%, 80%, 90%, and 100%) for 15 minutes. Finally, the samples were air dried, gold sputter coated and examined by SEM at accelerating voltage of 7 kV^31^.

#### pH values measurements

The pH values of culture liquid were measured by SG98 InLab pH meter (Mettler Toledo Int. Inc.) with a Expert Pro-ISM-IP67 probe. The fungal liquid was filtered through a 0.22 mm filter paper before the measurements.

#### High performance liquid chromatography

Organic acids were analyzed by HPLC (Agilent 1200). The column temperature of HPLC was 30 °C. The mobile phase consisted of basic ammonium and methanol with the ratio of 99:1. The phosphoric acid was applied to adjust the pH to 2.6 at a flow rate of 0.5 ml/min. Synthetic organic acids (analytical reagent) were used for calibration (Nanjing Chemical Reagent Co., LTD).

## Additional Information

**How to cite this article**: Li, Z. *et al.* A study of organic acid production in contrasts between two phosphate solubilizing fungi: *Penicillium oxalicum* and *Aspergillus niger*. *Sci. Rep.* **6**, 25313; doi: 10.1038/srep25313 (2016).

## Figures and Tables

**Table 1:** Variation of acidity and biomass of the fungi in the culture medium with various pH values (N = 3).

Original pH		6.5	5.5	4.5	>3.5	2.5	1.5
NJDL-03	Final pH	3.60 ± 0.07	3.77 ± 0.06	3.84 ± 0.01	3.46 ± 0.01	2.35 ± 0.00	1.54 ± 0.00
	Biomass	465.15 ± 5.36	481.5 ± 0.18	497.28 ± 2.51	472.83 ± 5.36	N.A.	N.A.
NJDL-12	Final pH	2.40 ± 0.03	2.35 ± 0.05	2.41 ± 0.03	2.28 ± 0.03	2.16 ± 0.01	1.53 ± 0.01
	Biomass	310.50 ± 11.38	324.57 ± 5.55	310.58 ± 5.36	309.30 ± 3.59	389.13 ± 14.76	333.80 ± 15.00

**Table 2:** Types and quantities of the secreted organic acids in the different culture medium (mg/L) (N = 3).

Strains	Organic acid type	pH					
		6.5	5.5	4.5	3.5	2.5	1.5
NJDL-03	oxalic acid	902 ± 3	478 ± 18	167 ± 35	–	N.A.	N.A.
	formic acid	1546 ± 174	1243 ± 123	591 ± 120	34 ± 3	N.A.	N.A.
	tartaric acid	1188 ± 155	769 ± 133	483 ± 67	55 ± 4	N.A.	N.A.
	malic acid	323 ± 9	302 ± 13	203 ± 6	77 ± 9	N.A.	N.A.
	citric acid	530 ± 29	459 ± 35	433 ± 24	229 ± 53	N.A.	N.A.
	Total	4489	3251	1877	395	N.A.	N.A.
NJDL-12	oxalic acid	2353 ± 128	2490 ± 130	1525 ± 79	1483 ± 112	701 ± 29	437 ± 29
	formic acid	7656 ± 350	8630 ± 441	5766 ± 324	5637 ± 277	3211 ± 154	1710 ± 95
	malic acid	–	–	493 ± 12	472 ± 17	252 ± 6	294 ± 4
	acetic acid	–	–	–	–	–	114 ± 2
	citric acid	322 ± 24	470 ± 37	372 ± 20	403 ± 17	960 ± 86	1841 ± 237
	Total	10331	11590	8156	7995	5124	4396

-: under detection limit.
